# Characterization of Three Ocular Clinical Isolates of *P. aeruginosa*: Viability, Biofilm Formation, Adherence, Infectivity, and Effects of Glycyrrhizin

**DOI:** 10.3390/pathogens6040052

**Published:** 2017-10-24

**Authors:** Xudong Peng, Sandamali A. Ekanayaka, Sharon A. McClellan, Ronald P. Barrett, Kerry Vistisen, Linda D. Hazlett

**Affiliations:** 1 Department of Anatomy and Cell Biology, Wayne State University School of Medicine, 540 E. Canfield Avenue, Detroit, MI 48201, USA; GF9681@wayne.edu (X.P.); sekanaya@med.wayne.edu (S.A.E.); smcclell@med.wayne.edu (S.A.M.); rbarrett@med.wayne.edu (R.P.B.); kvistise@med.wayne.edu (K.V.); 2 Department of Ophthalmology, The Affiliated Hospital of Qingdao University, Qingdao 266071, China

**Keywords:** *Pseudomonas aeruginosa*, keratitis, mice, glycyrrhizin

## Abstract

We selectively characterized three isolates from *Pseudomonas aeruginosa* keratitis patients and how glycyrrhizin (GLY) affected them. Type III toxins were determined using polymerase chain reaction (PCR). Minimum Inhibitory Concentration (MIC) of GLY and assays for its effects on: time kill, bacterial permeability, and biofilm/adhesion were done. In vivo, C57BL/6 (B6) mice were treated topically with GLY after G81007 infection. Clinical score, photography with a slit lamp and RT-PCR were used to assess treatment effects. Isolates expressed *exoS* and *exoT*, but not *exoU*. MIC for all isolates was 40 mg/mL GLY and bacteriostatic effects were seen for G81007 after treatment using time kill assays. From viability testing, GLY treatment significantly increased the number of permeabilized bacteria (live/dead assay). Isolates 070490 and G81007 formed more biofilms compared with R59733 and PAO1 (control). GLY-treated bacteria had diminished biofilm compared with controls for all isolates. GLY reduced adherence of the G81007 isolate to cultured cells and affected specific biofilm associated systems tested by reverse transcription PCR (RT-PCR). In vivo, after G81007 infection, GLY treatment reduced clinical score and messenger RNA (mRNA) expression of IL-1β, TNF-α, CXCL2 and HMGB1. This study provides evidence that GLY is bacteriostatic for G81007. It also affects biofilm production, adherence to cultured cells, and an improved keratitis outcome.

## 1. Introduction

Infection with *Pseudomonas aeruginosa* (*P. aeruginosa*), an opportunistic, Gram-negative bacterium, is often associated with microbial keratitis, especially in extended-wear contact lens users [1,2,3]. Pseudomonas keratitis develops rapidly and elicits an acute inflammatory response in cornea, which contributes to eradication of the bacterium. However, if inflammation is uncontrolled, this leads to significant corneal damage and loss of vision. Intensive antibiotic therapy is used to treat the disease, but with the increase in multidrug resistance, treatment is more difficult. The pathogenesis of *P. aeruginosa* is related to the production of a large number of secreted and cell-associated virulence factors including toxins and enzymes [4]. Toxins are often secreted through different types of protein secretion systems, including types I, II, III, V and VI [5]. *P. aeruginosa* may possess one or more of the genes *exoS*, *exoT* and *exoU,* which code for the cytotoxins ExoS, ExoT, and ExoU, secreted through the type III secretion system. These cytotoxins promote bacterial evasion of the host immune response, the dissemination of the organism from the infection site, and inhibition of DNA synthesis, leading to host cell death [6,7,8,9]. In addition, biofilm formation allows the bacteria to encase itself in a protective polymer matrix of polysaccharides, polypeptides, and extracellular DNA, and persist within the host during infections [10,11]. Biofilm formation protects the bacteria from the host immune response, facilitates microbial survival in hostile environments, and enhances antibiotic resistance [12]. Because of the latter, it appears advantageous to develop alternative or adjunctive therapeutics for bacterial infections [13,14]. High mobility group box 1 (HMGB1), a prototypic alarmin, enhances inflammation in many diseases [15,16,17,18] by binding to cell surface receptors, inducing the release of pro-inflammatory molecules that amplify an inflammatory cascade [19,20]. Recently, we described a strong correlation between HMGB1 and the severity of *P. aeruginosa* keratitis. In a prophylactic study, we demonstrated that reducing its level improved the disease outcome after infection with an invasive clinical keratitis isolate [21]. HMGB1 is a target for glycyrrhizin (GLY), the main triterpenoid saponin extracted from *Glycyrrhiza glabra*. Both GLY and its biologically active metabolite, glycyrrhetinic acid, are known for their anti-inflammatory properties. The structure of GLY was identified in 1989 as 3-*O*-β-d-glucurono-pyranosyl-(1–2)-β-d-glucurono-pyranosyl-glycyrrhetinic acid. GLY is formed from the Ca^2+^ and K+ salts of glycyrrhizic acid. The latter is the diglucuronide of 18β-glycyrrhetinic acid [22]. GLY binds HMGB1 and counteracts its chemokine and cytokine mediated inflammatory cascade [23,24]. Licorice root derivative also possesses antibacterial activity on skin [25], and in the respiratory [26] and urinary systems [27]. An extract of GLY also exhibits numerous pharmacological effects and has been used in the clinical management of chronic hepatitis [28]. We performed the current study to further determine the therapeutic effects of GLY on additional keratitis isolates and whether it was effective at reducing bacterial virulence factors. Evidence shows that GLY treatment reduced bacterial growth, viability, adhesion to lung and human corneal epithelial cells, and biofilm formation in vitro, as well as reducing keratitis and pro-inflammatory molecules in an in vivo animal model. 

## 2. Results

### 2.1. Gel Electrophoresis of PCR Products for Clinical Isolates

**Figure 1:**
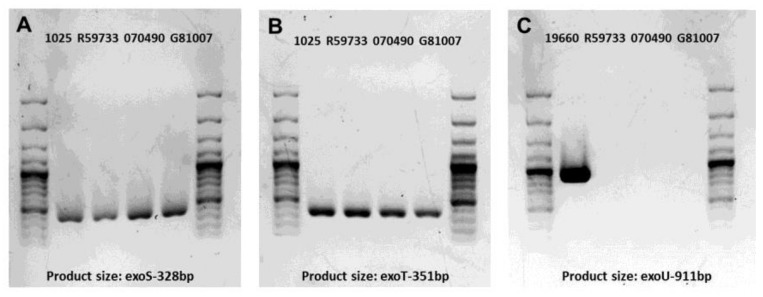
Gel electrophoresis of polymerase chain reaction (PCR) products of the cytotoxin genes among *P. aeruginosa* keratitis isolates. Amplification of the *exoS*, *exoT* and *exoU* genes (**A**–**C**). (**A**,**B**) clinical isolate KEI 1025 produces *exoS* (**A**) and *exoT* (**B**) and served as a positive control for the three isolates tested. (**C**) ATCC strain 19660 was the positive control for the *exoU* gene which could not be amplified in any of the three clinical isolates tested.

In order to begin to characterize the three clinical isolates of *P. aeruginosa*, we tested for type III secretion toxin encoding gene amplification patterns. These amplification patterns are shown in Figure 1A–C. All three clinical isolates (Kresge Eye Institute-KEI, R59733, 070490 and G81007) showed amplification of *exoS* and *exoT* (Figure 1A,B). None of the isolates showed amplification of the *exoU* gene and therefore were considered not cytotoxic (Figure 1C). Positive controls (KEI 1025 and ATCC 19660) showed amplification of the genes tested.

### 2.2. Minimum Inhibitory Concentration (MIC): Effect of GLY

**Figure 2:**
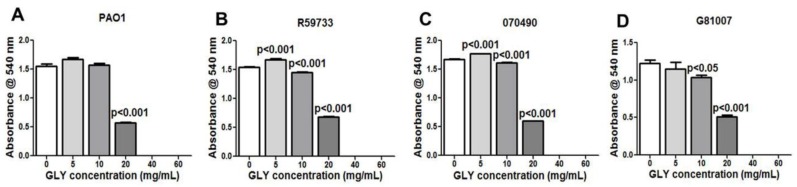
Minimum inhibitory concentration (MIC) of glycyrrhizin (GLY). Treatment with GLY inhibited visible bacterial growth in a concentration dependent manner for all strains tested (**A**–**D**) and completely inhibited visible growth at 40 and 60 mg/mL concentrations. All data are mean ± SEM and were analyzed using an unpaired, two-tailed Student’s *t*-test (*n* = 3/group/isolate).

Moving from characterizing type III secretion toxin encoding gene amplification patterns, we next began to test the MIC of GLY for each isolate and whether it had similar effects for each. MIC was determined for PAO1 (Figure 2A, control) and clinical isolates R59733 (Figure 2B), 070490 (Figure 2C), and G81007 (Figure 2D). Absorbance values measuring visible bacterial growth for PAO1 (Figure 2A) and the clinical isolates (Figure 2B–D) were completely inhibited at 40 and 60 mg/mL GLY. At 5 mg/mL, a slight increase in absorbance was seen for PAO1 (Figure 2A) (not significant), but significant for R59733 (Figure 2B) and 070490 (Figure 2C) (both = *p* < 0.001). At 10 mg/mL, significant reduction in absorbance was seen for R59733 (Figure 2B), 070490 (Figure 2C) (both = *p* < 0.01) and G81007 (Figure 2D) (*p* < 0.05); PAO1 was similar to 0 mg/mL GLY. At 20 mg/mL, absorbance values were significantly reduced for all groups (all = *p* < 0.001) (Figure 2A–D).

### 2.3. Time Kill Assay

**Figure 3:**
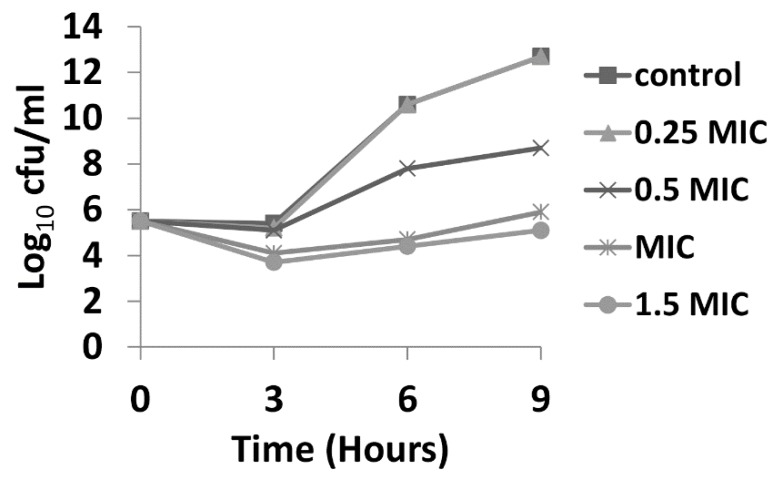
Time kill curves for isolate G81007 exposed to 0.25, 0.5, 1, and 1.5 × MIC of GLY. Bacteriostatic effects (<3log_10_ reduction in colony counts) were seen after 3, 6, and 9 h of incubation when compared with the starting inoculum at 0 h.

Time kill assays were performed over a period of 9 h, with bacteria being exposed to 0.25, 0.5, 1.0, and 1.5 × MIC of GLY (MIC = 40 mg/mL). Figure 3 shows time kill curves for G81007 (selected as a representative of the three clincal isolates that showed similar MIC values). At all concentrations × MIC of GLY, a decrease (less than 3log_10_ cfu/mL) in colony counts after 3 h was observed. At 6 h, only exposure to MIC and 1.5 × MIC showed similar bacteriostatic effects. At 9 h bacteriostatic activity was seen only for 1.5 × MIC.

### 2.4. Live/Dead Baclight Assay 

**Figure 4:**
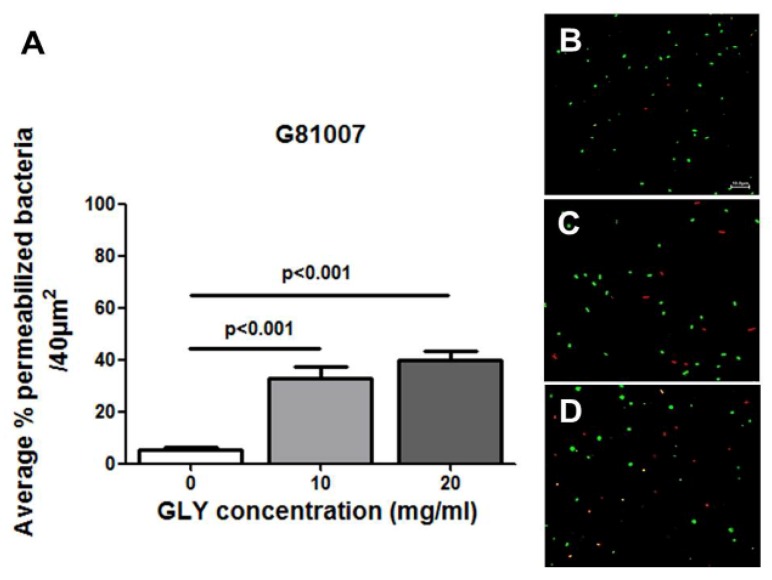
Live/Dead Baclight assay (**A**–**D**). Quantitatively, the average percentage of permeabilized (red) bacteria was increased with increased concentration of GLY (**A**). A confocal microscope was used to provide microscopic images for quantitation of live (green) vs. permeabilized (red) bacteria. These are shown without (**B**) and after GLY [10 mg/mL (**C**) and 20 mg/mL (**D**)] pretreatment of the bacteria. All data are mean ± SEM and were analyzed using a Bonferroni’s multiple comparison test (*n* = 16 areas /group). Bar (shown only in **B**) is at 10 μm.

A Live/Dead Baclight assay was used to selectively test isolate G81007 with or without GLY treatment. Figure 4 shows quantitative data from confocal images (Figure 4B–D) used to quantitate permeabilized (red) vs. live (green) bacteria. Figure 4A shows that with increased GLY concentration (both 10 and 20 mg/mL), the number of permeabilized (red) bacteria are significantly increased (*p* < 0.001) compared with control (no GLY). 

### 2.5. Biofilm Formation and Exposure to GLY

**Figure 5:**
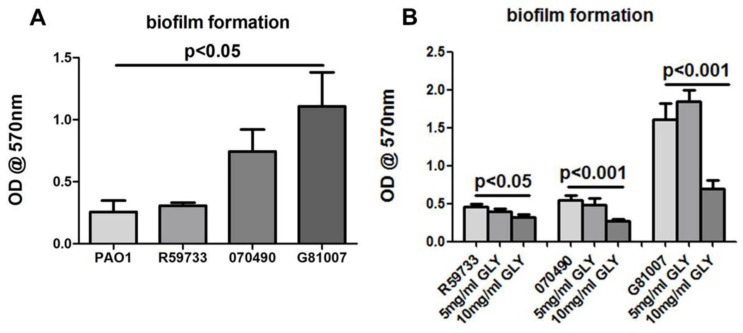
Effect of GLY on biofilm formation of the three clinical isolates. The optical density (OD) values of biofilm formation produced by 070490 and G81007 increased above the control (PAO1), but was only significant for G81007 (**A**). There was no significance between R59733 or 070490 and PAO1 (**A**). Production of biofilm after treatment of the bacterial isolates with GLY is shown (**B**). Biofilm formation activity for all three isolates was inhibited significantly at 10 mg/mL GLY when compared with no GLY treatment. For the G81007 isolate, a slight but not significant increase in biofilm production was seen at 5 mg/mL. All data are mean ± SEM and were analyzed using a two tailed student’s *t* test (*n* = 6/group/strain).

Screening of in vitro biofilm production showed that all three clinical isolates are biofilm producers (Figure 5A,B). For this assay, ATCC strain PAO1, which produces a non-mucoid biofilm, was used as a positive control (Figure 5A). Biofilm production by isolate R59733 was not statistically significant when compared to the non-mucoid biofilm producing strain PAO1. In contrast, an increase in biofilm production was noted for isolates 070490 and G81007 compared to PAO1, but only the latter was significant (*p* < 0.05) (Figure 5A). Treatment with GLY decreased the detected attached biomass for all three isolates significantly at 10 mg/mL (R59733 = *p* < 0.05 and both 070490 and G81007 = *p* < 0.001) as shown in Figure 5B. Note that for G81007, a slight, but not significant increase in biofilm formation was detected at 5 mg/mL GLY, which was not seen for the other two isolates.

### 2.6. Bacterial Adherence Assay

**Figure 6:**
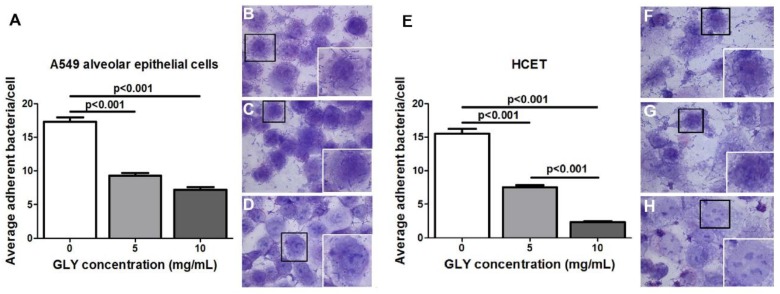
G81007 adherence to A549 alveolar epithelial (**A**–**D**) and HCET (**E**–**H**) cells. The average number of adherent bacteria per cell was decreased at both concentrations of GLY (**A**,**E**). Adherent bacteria are shown in images taken using a bright field microscope before (**B**,**F**) and after GLY [5 mg/mL (**C**,**G**) and 10 mg/mL (**D**,**H**)] treatment. An inset is shown in (**B**–**D**,**E**–**H**), and these are an enlargement of the cells in each photomicrograph that are outlined by a black box. All data are mean ± SEM and were analyzed using the Bonferroni’s multiple comparison test (*n* = 100 cells/group). Magnification = ×20 μm, inset magnification = ×40 μm.

Since G81007 produced the greatest amount of biofilm of the three isolates, A549 lung epithelial cells and transformed human corneal epithelial cells (HCET) were used to test the ability of the isolate to adhere to these cells (first step in biofilm formation). The data are shown in Figure 6A–H. Bacteria were untreated (Figure 6B–F) or treated with GLY (Figure 6C,D,G,H) just before application to the lung (Figure 6A–D) or human corneal epithelial (Figure 6E–H) cells, and incubated for 3 h. Wright-Giemsa staining revealed bacteria adherent to A549 alveolar epithelial and human corneal epithelial cells in both control (Figure 6B,F) and GLY-treated (Figure 6C,D,G,H) groups. The clinical isolate G81007 combined with GLY immediately before application to the cultured cells had a significantly decreased number of adherent bacteria (*p* < 0.001) at both 5 (Figure 6C,G) and 10 (Figure 6D,H) mg/mL concentrations. With increased amounts of GLY, the average percentage of adhered bacteria to the cultured cells was decreased (Figure 6A,E) compared to control. There also was a significant reduction in adherent bacteria for HCET cells treated with 10 vs. 5 mg/mL GLY (*p* < 0.001, Figure 6E).

### 2.7. RT-PCR: mRNA Expression of Bacterial Factors Implicated in the Regulation of P. aeruginosa Biofilm Formation After GLY Treatment

**Figure 7:**
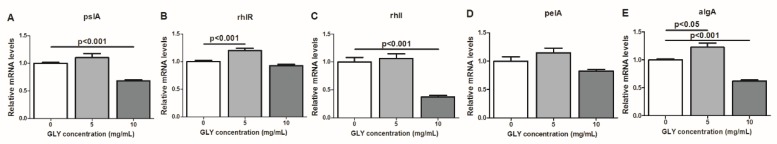
Expression of bacterial factors involved in biofilm production (**A**–**E**). After treatment of the bacteria with GLY (0, 5 and 10 mg/mL) for 3 h, mRNA expression levels for bacterial factors *rhlR* (**B**) and *algA* (**E**) were significantly elevated at 5 mg/mL. Genes *rhlI*, *pslA* and *pelA* were elevated, but not significant compared with control. At 10 mg/mL, bacterial factors *pslA* (**A**), *rhlI* (**C**) and *algA* (**E**) mRNA expression levels were significantly reduced; *rhlR* (**B**) and *pelA* (**D**) mRNA levels were also reduced, but not significant. Data are mean ± SEM analyzed using a two tailed student’s *t* test. *n* = 3/group/time.

As a follow up to the above experiment, mRNA expression of bacterial factors that regulate biofilm formation were similarly tested (Figure 7A–E) in a separate experiment. Bacteria were incubated with GLY (0, 5 and 10 mg/mL) for 3 h, and bacterial systems and factors tested included: *pslA*, (Figure 7A) a polysaccharide for biofilm stability, *rhlR* (Figure 7B) and *rhlI* (Figure 7C), members of the quorum sensing system, *pelA* (Figure 7D) and *algA* (Figure 7E), involved in exopolysaccharide production and c-di-GMP pool regulation. GLY at 5 mg/mL modestly, but significantly elevated *rhlR* (*p* < 0.001) and *algA* (*p* < 0.05) (Figure 7B,E) with no significance compared to controls for the other genes tested. GLY at 10 mg/mL modestly, but significantly reduced mRNA levels for *pslA* (Figure 7A), *rhlI* (Figure 7C), and algA (Figure 7E) vs. control. 

### 2.8. Infectivity of Isolate G81007 and GLY Treatment 

**Figure 8:**
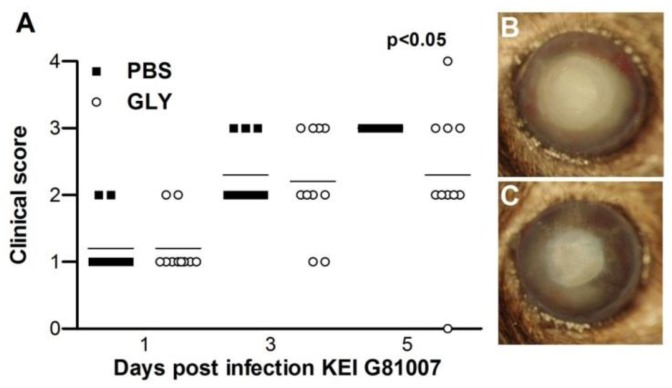
Treatment with GLY after infection with isolate G81007. Clinical scores (**A**) were reduced significantly at 5, but not at 1 and 3 days post infection (p.i.) in GLY versus PBS-treated mice (*n* = 5/group/time repeated once = 10/group). Photographs taken with a slit lamp at 5 days p.i. from (**B**) PBS- and (**C**) GLY-treated mice confirmed reduced opacity after GLY treatment. Horizontal lines indicate median values. Data were analyzed using a nonparametric Mann-Whitney U-test. Photographs taken with a slit lamp are at a magnification of ×8.

G81007 was tested in an in vivo mouse model of keratitis. Severity of disease was graded (clinical scores, Figure 8A) and photographs were taken with a slit lamp (Figure 8B,C) following infection. Treatment with GLY (20 mg/mL) vs. phosphate-buffered saline (PBS) (Figure 8A) showed reduced clinical scores that were significant at 5 days post infection (p.i.) (*p* < 0.05), but not at 1 or 3 days p.i. Photographs taken with a slit lamp confirmed less opacity in the GLY (Figure 8C) compared with the PBS (Figure 8B)-treated eye. 

### 2.9. RT-PCR: GLY Treatment after G81007 Infection

**Figure 9:**
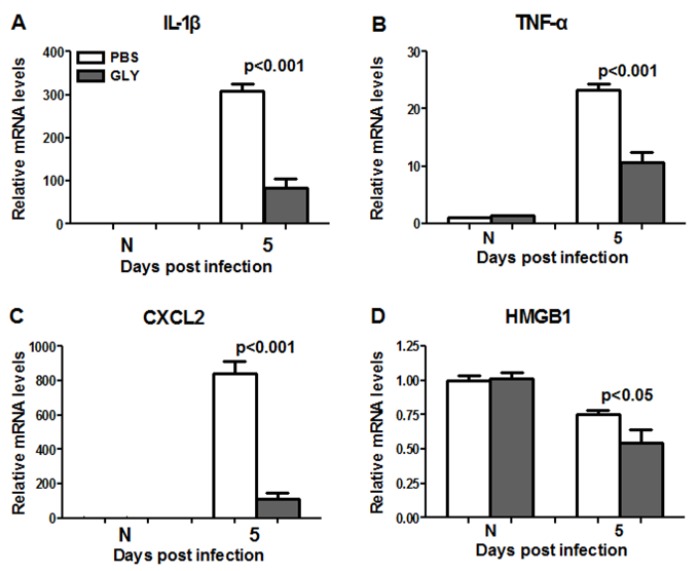
Real-time RT-PCR after infection with isolate G81007. At 5 days p.i., corneal mRNA levels of IL-1β (**A**) TNF-α (**B**), CXCL2 (**C**) and HMGB1 (**D**) were reduced significantly in GLY vs. PBS treated corneas (concentration = 20 mg/mL). No difference between groups (**A**–**D**) for normal cornea (N) was seen. Data are mean ± SEM analyzed using a two tailed student’s *t* test (*n* = 5/group/time).

GLY significantly reduced mRNA levels for pro-inflammatory mediators IL-1β (Figure 9A, *p* < 0.001), TNF-α (Figure 9B, *p* < 0.001), CXCL2 (Figure 9C, *p* < 0.001), and HMGB1 (Figure 9D, *p* < 0.05) when compared with PBS at 5 days p.i. HMGB1 and TNF-α were detected in the normal (N) cornea (contralateral eye), but did not differ between groups. 

## 3. Discussion

*P. aeruginosa* is one of the common causes of microbial keratitis throughout the world [29], and is closely related to contact lens usage [30]. The ability of bacteria to develop resistance against multiple classes of antibiotics makes the condition more complicated to manage. Therefore, the further testing of GLY therapeutically as an alternative to antibiotic therapy, as done in this study, remains important. GLY, a glycoconjugated triterpene produced by *Glycyrrhiza glabra*, is a natural anti-inflammatory and bacteriostatic agent in clinical use. It possesses numerous pharmacological effects [31] and has been shown effective in animal models of keratitis (treatment was prophylactic) [21], colitis [32], sepsis [33], brain [34], and lung [26] injury. In addition, GLY has been used in clinical management to treat allergic conjunctivitis, blepharitis [20], and hepatitis B and C at high doses (up to 140 mg/day) [28,35] with no signs of adverse events or drug toxicity. HMGB1 is a nuclear component, but extracellularly, it serves as a signaling molecule involved in the pathogenesis of Pseudomonas keratitis [36], as well as other infectious diseases [19,37]. The strong correlation between HMGB1 and the pathogenesis of various infectious diseases [19] suggests that it may provide an optimum target for treatment and clinical use. In this regard, GLY directly binds to HMGB1 without interfering with its secondary structure. It inhibits extracellular HMGB1-mediated mitogenic and chemotactic functions. and has a weak inhibitory effect on intranuclear DNA-binding [24]. This has been tested prophylactically using a single clinical isolate and was efficacious [21]; however therapeutic testing was not explored thoroughly, nor were additional clinical isolates examined as in the current study.

*P. aeruginosa* uses a complex type III secretion apparatus, essentially a needle-like machine on the surface of the bacteria for the purpose of protein transport [4]. This complex regulon is divided into five parts: the needle complex that transports substrates from the bacterial cytosol to the external environment, proteins that translocate secreted proteins into host cells, proteins that regulate the secretion process, chaperone proteins that aid in secretion of their cognate partners, and proteins that are injected into host cells (effector proteins) [9]. These proteins modulate host cell functions, including signal transduction and cytoskeletal organization [7]. In the current study, we tested for amplification of type III genes *exoU*, *S* and *T* but did not test for *exoY*, as our goal was to distinguish invasive vs. cytotoxic organisms. We were able to amplify type III genes, *exoS* and *exoT*, for all three clinical isolates, but did not amplify *exoU*. We realize that there is a possibility that the genomic sequence for *exoU* in these clinical isolates is different enough from the reference ATCC strain, that primer annealing and PCR amplification do not effectively occur. However, others have reported success when using a single set of primers to amplify *exoU* [38,39,40]. In this regard, Berthelot et al. [40] have shown that expression of *exoS* and *exoU* were prevalent in 92 epidemiologically unrelated isolates of *P. aeruginosa*. ExoS and ExoT are bifunctional toxins exhibiting adenosine diphosphate (ADP)-ribosyltransferase and guanidine triphosphate (GTP)ase-activating activity [41]. ExoU [5] has phospholipase activity and disrupts eukaryotic membranes following its delivery into the cytoplasm. In fact, the presence of *exoS* and *exoU* differ noticeably between isolates, and even appear to be mutually exclusive [7]. In addition, a previous study has reported that the genes encoding the cytotoxins ExoS and ExoU are present as variable traits in *P. aeruginosa*, and their presence depends on the disease site [42]. 

In vitro, we tested the MIC of GLY, and absorbance data provided visible evidence that the compound itself exhibited activity against all three clinical isolates. These data agree with a previous study from this lab [21], and another study which tested *Glycyrrhiza glabra* against both Gram positive and Gram negative bacteria [43]. Both studies showed that the effects were concentration-dependent and suggest that GLY reduces bacterial load, and that this contributes to its anti-inflammatory effects. Time kill assays revealed that GLY at all concentrations × MIC, decreased (less than 3log_10_ cfu/mL) colony counts after 3 h. At 6 h, only exposure to MIC and 1.5 × MIC showed similar bacteriostatic effects. At 9 h, bacteriostatic activity was seen only for 1.5 × MIC. Because of solubility issues with GLY, we were not able to test beyond 1.5 × MIC and thus cannot provide information about potential bactericidal effects in vitro. Nonetheless, in vivo, we have shown the bactericidal effects of GLY using viable plate count. We reported that GLY reduced bacteria by about 4.5log_10_ cfu/mL in the keratitis model after infection with the clinical isolate KEI 1025 [21].

A Live/dead BacLight assay also was used to further evaluate bacterial viability [44,45]. A single clinical isolate, G81007, was tested before and after GLY treatment. Our data showed that GLY significantly increased the average percentage of permeabilized (red) bacteria with an increased concentration of GLY. These data were consistent with the MIC results and provided additional support confirming the antibacterial activity of GLY. However, there are issues with this assay. Others have pointed out that microscopic assessment of live/dead bacterial cells is oversimplified to either green labeled (live) or red labeled (dead) cells. In contrast, flow cytometry has shown that the staining of bacteria with SYTO9 and propidium iodide (PI) does not always produce these distinct two populations [46], that bacteria are in fact, permeabilized, and that intermediate states are also observed [46,47,48]. These latter are termed as “unknown”. In addition, it is likely that reduction in live bacteria was not just a viability issue, but also may be due to a reduction in biomass. 

To initiate an infection, bacteria must first colonize their host. Adhesion is a first step prior to invasion and/or secretion of toxins, thus it is a key event in bacterial pathogenesis [49]. *P. aeruginosa* produces several surface-associated adherence factors or adhesins which promote attachment to epithelial cell surface receptors or soluble proteins and contribute to the virulence of this pathogen [50]. The study reported herein used an in vitro adherence assay to test isolate G81007 binding to human A549 alveolar epithelial cells and to human corneal epithelial cells. It provides evidence that GLY (3 h incubation with bacteria and cultured cells) significantly reduces adherence of *P. aeruginosa* (clinical isolate G81007) in a concentration-dependent manner. Reduction in adherence could be due to several factors, including modest reduction in biomass at 3 h (Figure 3). Also, it is possible that the type IV pilus, which plays an important role in the adherence of *P. aeruginosa* to tracheal epithelia [51] and accounts for about 90% of adherence capability [50,52,53], might have been affected due to GLY interfering with receptor/adhesin interactions. Whether GLY could detach already adherent bacteria was not tested, but hypothetically this is likely, given its ability to reduce biomass and to permeabilize the bacterial cells.

Biofilm formation in *P. aeruginosa* has been considered an important determinant of pathogenicity during infections [11]. Biofilm production facilitates chronic bacterial infections and reduces the efficacy of antimicrobial therapy [54,55]. In order to determine biofilm activity, the three clinical isolates were tested using a colorimetric microtiter plate assay. Data showed that all of the isolates produced biofilm and G81007 was the strongest biofilm producer when compared with both other isolates and the non-mucoid biofilm producing strain, PAO1 which served as a control. Nonetheless, all isolates were tested further regarding the ability of GLY to inhibit biofilm production. GLY inhibited biofilm formation for all three isolates at 10 but not at 5 mg/mL concentration after the bacteria were incubated for 24 h with GLY. In addition, a slight increase in biofilm formation was seen at 5 mg/mL concentration only for the G81007 isolate. We hypothesize that reduction in biofilm formation after GLY treatment was the result of reduced biomass of the bacteria, but we do not know if other systems were affected.

To determine the mechanism by which GLY might inhibit biofilm formation further studies were initiated. GLY (incubated with equal amounts of the bacteria, as done for the adherence assay) was tested for its ability to attenuate virulence determinants *pelA* (Pel) and *rhlR*, *rhlI* (the rhl system) of *P. aeruginosa*. GLY treatment of G81007 followed by RT-PCR analysis revealed reduction in *pelA* (not significant) and statistically (perhaps not biologically) significant reduction of *algA* and *pslA*, polysaccharides that are required for stability of biofilm structure [56,57]. Alginate, a linear unbranched polymer composed of d-mannuronic acid and l-guluronic acid, contributes to the structural stability and protection of biofilms, as well as to the retention of water and nutrients [58]. Pel and Psl can serve as a primary structure scaffold for biofilm development and are involved at early stages of biofilm formation [59,60]. It is possible, but not yet tested, that GLY perturbed the stability of the biofilm by saponification of bacterial membranes [22], and thus may have interfered early on in disturbing Pel or Psl, and thus the formation of a primary scaffold for biofilm development. Certainly, without knowing which exopolysaccharide the strain uses for biofilm formation, we realize the difficulty of interpreting the functional significance of these intriguing data. 

GLY treatment (10 mg/mL) also reduced *rhlR* (not significant), and significantly reduced *rhlI*, members of the quorum sensing systems of *P. aeruginosa* that also regulate biofilm formation [61]. Disruption of this system, used for cell to cell communication, could potentially disrupt the ability of the bacteria to detect population density, as well as virulence factor production, motility, and biofilm formation [62,63]. The rhl system has been reported to intervene in *P. aeruginosa* biofilm formation [64] by enhancing Pel polysaccharide biosynthesis. Furthermore, transcription of the *pel* operon is reduced in a *rhlI* mutant, providing additional support for the function of this system. Hypothetically, disruption of the bacterial membrane due to the structural features of GLY [22], and decreased cell density, could compromise all of these functions.

GLY treatment (5 mg/mL for 3 h) of the G81007 isolate also modestly, but significantly, increased *rhlR* and *algA* and elevated all of the other genes tested, but this was not significant. These data are consistent with the slight elevation in biofilm production seen for this isolate at 5 mg/mL concentration of GLY (Figure 5B). Although hypothetical, the data appear consistent with others studies that have shown that at lower (sub-inhibitory) concentrations of antimicrobials (e.g., tobramycin), biofilm formation was induced [65]. Further testing of this novel hypothesis regarding a sub-inhibitory effect of GLY on the G81007 isolate would need to be performed. 

In vivo, preclinical animal studies of inflammatory diseases such as sepsis revealed that GLY inhibits expression, extracellular secretion, and cytokine activity of HMGB1 and other pro-inflammatory cytokines [37,66]. Most relevant to the current study, this laboratory provided the first thorough prophylactic evidence that GLY treatment is effective in bacterial keratitis induced by a noncytotoxic clinical isolate 1025. The reduction of HMGB1 appeared critical for optimum protection, as recombinant HMGB1 treatment with GLY reversed the latter’s protective effect. This proof of principle study established HMGB1 as a target for GLY treatment [21]. Because of the success of the prophylactic treatment using GLY in the above study [21], we tested whether therapeutic treatment was feasible and provided protection. At 6 h after infection with isolate G81007, topical treatment with GLY significantly reduced clinical scores, and these data were confirmed by photographs using a slit lamp. The latter showed reduced opacity at 5 days p.i. in the GLY vs. control treated mice. mRNA expression levels of HMGB1 and several pro-inflammatory molecules (IL-1β, CXCL2, TNF-α) also were reduced significantly, and agreed with data reported previously [21]. 

Collectively, these studies show that GLY reduces/inhibits bacterial growth, reduces early events of biofilm formation measured by adhesion to lung and human corneal epithelial cells, and affects genes contributing to biofilm formation, as well as reducing keratitis and pro-inflammatory molecules in vivo. 

## 4. Materials and Methods

### 4.1. Mice

Eight-week-old female C57BL/6 (B6) mice were purchased from the Jackson Laboratory (Bar Harbor, ME). Mice were housed in accordance with the National Institutes of Health guidelines. Animals were treated humanely and in compliance with the ARVO Statement for the Use of Animals in Ophthalmic and Vision Research.

### 4.2. Bacterial Culture and Infection

*P. aeruginosa* isolates including: clinical isolates KEI 1025, R59733, 070490 and G81007 (Kresge Eye Institute, Detroit, MI, USA); strain 19660 and PAO1 (American Type Culture Collection [ATCC] Manassas, VA, USA) were grown in peptone tryptic soy broth (PTSB) medium at 37 °C in a rotary shaker water bath at 150 g for 18 h to an optical density (measured at 540 nm) between 1.3 and 1.8. Bacteria were pelleted by centrifugation at 5500 *g* for 10 min, washed once with sterile saline, recentrifuged, resuspended, and diluted in sterile saline (0.85%, pH = 7.4). For in vivo infection, mice were anesthetized (using anhydrous ethyl ether) and placed beneath a stereoscopic microscope at ×40 magnification. The left cornea was scarified by making three 1 mm incisions with a sterile 25^5/8^-gauge needle. A 5 μL aliquot of *P. aeruginosa* (G81007, 1 × 10^7^ cfu/μL) was applied topically to the wounded cornea. Similar testing of the other two isolates provided inconsistent (mild) infection and were not used to test the in vivo effects of GLY.

### 4.3. Ocular Response to Bacterial Infection

Corneal disease was scored at 1, 3, and 5 days postinfection (p.i.). Clinical scores were designated as: 0, clear or slight opacity, partially or fully covering the pupil; +1, slight opacity, fully covering the anterior segment; +2, dense opacity, partially or fully covering the pupil; +3, dense opacity, covering the entire anterior segment; and +4, corneal perforation or phthisis. Clinical scores were used to statistically compare disease severity and accompanied by photographs taken with a slit lamp (5 days p.i.) to illustrate disease [67].

### 4.4. GLY Treatment

Infected eyes of B6 mice (*n* = 5/group/time, repeated once) were treated with 5 μL of GLY 20 mg/mL (Sigma-Aldrich Corp., St. Louis, MO, USA) or PBS (control) topically. Treatment began at 6 h p.i. and then twice per day at 1–4 days p.i. Dosage was based on reports for drug concentrations of GLY used by other laboratories [68] and as reported before from this laboratory [21]. PBS was used similarly as the control [21].

### 4.5. Amplification of Virulence Genes Encoded by Type III Secretion System

20 μL of tissue buffer (0.25% SDS + 0.05 M NaOH) was mixed with a single colony of a bacterial isolate, then the mixture was incubated at 95 °C for 10 min. After incubation, the mixture was centrifuged for 1 min in 13,000× *g* and finally 180 μL of diethylpyrocarbonate (DEPC)-treatedwater was added. Virulence genes *exoS*, *exoT* and *exoU* (positive controls included KEI 1025 and ATCC 19660) were amplified by the PCR method using specific primers shown in Table 1. Each PCR reaction was done in a total volume of 20 μL as follows: 2 μL of template DNA, 0.5 μL of each primer (10 μM), 7 μL DEPC-treated water, and 10 μL of KAPA2G Fast HS Genotyping Mix (Kapa Biosystems, Wilmington, MA, USA). The PCR condition was carried out as follows: initial denaturation step (at 95 °C for 3 min), followed by 35 to 40 cycle repetitions of denaturation (15 s at 95 °C), annealing (15 s at 60 °C), and extension (15 s at 72 °C) with a final extension at 72 °C for 10 min [69,70]. PCR products were analyzed by electrophoresis in 1% agarose gels with 4 μL GreenGloTM (Denville Scientific Inc., Holliston, MA, USA) added to the melted agarose when it cooled to 55 °C for 70–80 min at 100 V. Finally the gel was scanned on a Fluor Chem E imaging system (Cell Biosciences, Santa Clara, CA, USA).

### 4.6. Real Time RT-PCR 

Normal (uninfected) and infected (G81007) corneas were removed at 5 days p.i. from B6 mice after GLY- or PBS-treatment. In a separate experiment, isolate G81007 was treated with serial dilutions of GLY (0, 5 and 10 mg/mL) for 3 h. For each cornea and bacterial pellet (1.5 mL of each bacterial culture/pellet), total RNA was isolated (RNA STAT-60; Tel-Test, Friendswood, TX, USA) according to the manufacturer’s instructions and as reported before. Quantity and quality of extracted RNA were determined using a NanoDrop ND-1000 Spectrophotometer (ThermoFisher Scientific, Waltham, MA, USA) by measuring absorbance at 230, 260 and 280 nm. 1 μg of each RNA sample was reverse transcribed using Moloney-murine leukemia virus (M-MLV) reverse transcripts (Invitrogen, Carlsbad, CA, USA) to produce a cDNA template for the PCR reaction. Complementary (c) DNA products were diluted 1:25 with DEPC-treated water. A 2 μL aliquot of diluted cDNA was used for the real-time RT-PCR reaction with real-time SYBR green/fluorescein PCR master mix (Bio-Rad Laboratories, Richmond, CA, USA) and primer concentrations of 10 μM (total 10 μL reaction volume). After a preprogrammed hot start cycle (3 min at 95 °C), the parameters used for PCR amplification were: 15 s at 95 °C and 60 s at 60 °C with the cycles repeated 45 times. Optimal conditions for PCR amplification of cDNA were established using routine methods and all procedures are as described before [21,36]. Levels of corneal mRNA of HMGB1, IL-1β, TNF-α, and CXCL2, and bacterial genes *pslA*, *rhlR*, *rhlI*, *pelA*, and *algA* were tested by real-time RT-PCR (CFX Connect real-time PCR detection system; Bio-Rad Laboratories). The fold-differences in gene expression were calculated relative to housekeeping genes; β-actin (for cornea) and *rpoD* (for bacterial genes) [71] and then expressed as the relative mRNA concentration (control sample set to value of 1) ± SEM. The primer pair sequences used for real-time RT-PCR are shown in Table 2 and Table 3.

### 4.7. Minimum Inhibitory Concentration (MIC)

Clinical isolates were prepared as described above and MIC was determined as reported before [21,72] with the following modifications. Serial dilutions of GLY (5 mL/tube) were prepared in PTSB (0–60 mg/mL) and 10 μL of each bacterial culture (adjusted to 1.5 × 10^8^ cfu/mL using the 0.5 McFarland standard) was added to each tube and incubated at 37 °C for 18 h. Visible bacterial growth at each GLY concentration was examined by spectrophotometry (absorbance value) at 540 nm. MIC was assigned as the lowest GLY concentration that inhibited visible bacterial growth.

### 4.8. Time Kill Assay

Time kill assays are commonly used with MIC to evaluate bactericidal/bacteriostatic activity of an antimicrobial agent alone or in combination [73,74]. These assays provide reliable data on antimicrobial tolerance and also best correlate with cures in animal models [75]. The conventional colony counting based method is the most used assay [75] and was used for this study. For this, a microbial inoculum was exposed to GLY (concentration based on MIC) at time zero and grown for 9 h. Controls were similarly treated but with no GLY. At selected time points (0, 3, 6 and 9 h), aliquots were withdrawn from the inoculum, diluted and plated in triplicate.

All plates were incubated for 18 h and colonies counted. Bactericidal activity is defined as a reduction in colony count ≥3log_10_ cfu/mL, and bacteriostatic activity is defined as a decrease in colony count <3log_10_ cfu/mL [75]. The reduction in colony count is calculated compared with the colony count of the inoculum at time zero. 

### 4.9. Bacterial Adherence Assay 

Isolate G81007 was prepared as described above and bacterial growth examined as described before [21]. Briefly, bacterial cultures were grown overnight at 37 °C in PTSB for 18 h. Bacterial suspensions were prepared in sterile saline, adjusted to a concentration of 1.5 × 10^8^ cfu/mL using the 0.5 McFarland standard. A549 human type II alveolar epithelial cells (ATCC #CCL-185) and transfected human corneal epithelial cells (HCET, cell line 10.014 pRSV-T, gift of Dr. Gabriel Sosne) (each cell line = 2 × 10^5^ cells) in 1 mL of complete media were seeded onto chambered slides (4/slide) and incubated overnight. Cell chambers were washed twice with D-PBS, then 1 mL fresh media was added without antibiotics. Bacteria (washed and reconstituted) were combined with GLY (0, 5 and 10 mg/mL) immediately before application to the chambered slides in a volume equal to 10 multiplicity of infection (MOI)/cell. Each slide was incubated for 3 h at 37 °C under aerobic conditions. Then slides were washed three times with sterile PBS. After air drying, each slide was stained with 0.5 mL Wright-Giemsa stain (Sigma) for 30 s, and 0.5 mL PBS was added for another min. Then chambers were gently decanted and washed with PBS. After air drying, coverslips were mounted to the chambered slide with permount. The number of viable bacteria which adhered to the surface of 100 A549 and/or HCET cells were counted, averaged, and expressed as number of adherent bacteria/cell. All specimens were observed by bright field microscopy (DM4000B; Leica Microsystems, Exton, PA, USA) and photographed using oil immersion (100× objective) (*n* = 100 cells/group). 

### 4.10. Live/Dead BacLight Bacterial Viability

GLY (0, 10 and 20 mg/mL) was prepared in PTSB in sterile tubes, and 10 μL of G81007 (washed and reconstituted in saline) was added and incubated at 37 °C for 18 h. A 1 mL suspension of GLY treated isolate G81007 was placed into a microfuge tube (washed and reconstituted in saline) and stained with the Live/Dead BacLight Bacterial Viability L7012 solution (Molecular Probes, Waltham, MA, USA) reagents per the manufacturer’s directives. Equal volumes of component A and B were combined and mixed thoroughly in a microfuge tube. 3 μL of the dye mixture was added for each mL of the bacterial suspension and incubated at room temperature in the dark for 15 min. 5 μL of the stained bacterial suspension was placed on a slide and cover slipped. All specimens were observed by confocal microscopy (TCSSP8; Leica Microsystems, Exton, PA, USA). Bacteria incubated similarly but with PBS and no GLY served as a control. For quantitation, three representative confocal micrographs were analyzed essentially as described by Senoo et al. [76]. Briefly, the total number of green or red bacteria without GLY treatment were counted in four 40 μm^2^ areas of each micrograph and, compared to the total number of bacteria which were treated with 10 or 20 mg/mL of GLY (16 areas/group). Viable cells exhibited green fluorescence, while cells with membrane damage (permeabilized) fluoresced red. The number of live or permeabilized bacteria were enumerated, averaged, and expressed as a percent of the total number of bacteria.

### 4.11. Biofilm Formation and Exposure to Glycyrrhizin

Quantitative determination of biofilm forming capacity was tested (R59733, 070490 and G81007) by a colorimetric microtiter plate assay [77]. Briefly, bacterial cultures were grown overnight at 37 °C in PTSB for 18 h. Bacterial suspensions were washed and prepared in sterile saline and adjusted to a concentration of 1.5 × 10^8^ cfu/mL using the 0.5 McFarland standard. 90 μL of PTSB with serial dilutions of GLY (0, 5 and 10 mg/mL) and 10 μL of the bacterial suspension was added to each well of a microtiter plate (in triplicate) [77]. Medium was added as the negative control for each plate. Subsequent to an incubation period of 24 h at 37 °C in aerobic conditions without shaking, wells were gently decanted and rinsed three times with 200 μL of sterile PBS (pH 7.2). After air drying, biofilms were fixed with 100 μL of 99% methanol per well for 20 min, dried and stained with 100 μL per well of 2% crystal violet (Sigma) for 15 min. Unbound dye was rinsed with water. After air drying, dye bound to biofilm formed on the chamber was released with 100 μL of 33% acetic acid per well for 30 min at room temperature, without shaking. OD of each well was measured at 570 nm using a microtiter plate reader. 

### 4.12. Statistical Analysis

The difference in clinical score between two groups was tested by the Mann-Whitney U test. For comparison of three or more groups (bacterial viability and adherence assay), a 1-way ANOVA followed by the Bonferroni’s multiple comparison test (GraphPad Prism) was used for analysis. An unpaired, two-tailed Student’s *t*-test was used to determine the statistical significance of the MIC, biofilm formation and RT-PCR data. For each test, *p* < 0.05 was considered significant and data was shown as mean ± SEM. All experiments were repeated at least once to ensure reproducibility.

## Figures and Tables

**Table 1:** Primers used for the amplification of different cytotoxin genes among *P. aeruginosa* isolates.

Cytotoxin Gene	Nucleotide Sequence (5′-3′)	Product Size (bp)	Reference
*exoS*	F-ATC CTC AGG CGT ACA TCC	328	[69]
	R-ACG ACG GCT ATC TCT CCA C		
*exoT*	F-AGA ACC CGT CTT TCG TGG CTG AGT T	351	[69]
	R-CAG CTC GCT CGC CTT GCC AAG T		
*exoU*	F-CCT TAG CCA TCT CAA CGG TAG TC	911	[70]
	R-GAG GGC GAA GCT GGG GAG GTA		

F, forward; R, reverse.

**Table 2:** Nucleotide sequence of the specific primers used for PCR amplification.

Gene	Nucleotide Sequence	Primer	GenBank
*β-actin*	5′-GAT TAC TGC TCT GGC TCC TAG C-3′	F	NM_007393.3
	5′-GAC TCA TCG TAC TCC TGC TTG C-3′	R	
*HMGB1*	5′-TGG CAA AGG CTG ACA AGG CTC-3′	F	NM_010439.3
	5′-GGA TGC TCG CCT TTG ATT TTG G-3′	R	
*IL-1β*	5′-CGC AGC AGC ACA TCA ACA AGA GC-3′	F	NM_008361.3
	5′-TGT CCT CAT CCT GGA AGG TCC ACG-3′	R	
*CXCL2*	5′-TGT CAA TGC CTG AAG ACC CTG CC-3′	F	NM_009140.2
	5′-AAC TTT TTG ACC GCC CTT GAG AGT GG-3′	R	
*TNF-α*	5′-ACC CTC ACA CTC AGA TCA TCT T-3′	F	NM_013693.2
	5′-GGT TGT CTT TGA GAT CCA TGC-3′	R	

F, forward; R, reverse.

**Table 3:** Nucleotide sequence of the specific primers used for PCR amplification.

Gene	Nucleotide Sequence	Primer	Gen ID
*rpoD*	5′-CAT CCG CAT GAT CAA CGA CA-3′	F	882038
	5′-GAT CGA TAT AGC CGC TGA GG-3′	R	
*pslA*	5′-AAG ATC AAG AAA CGC GTG GAA T-3′	F	879717
	5′-TGT AGA GGT CGA ACC ACA CCG-3′	R	
*rhlR*	5′-CTG GGC TTC GAT TAC TAC GC-3′	F	878968
	5′-CCC GTA GTT CTG CAT CTG GT-3′	R	
*rhlI*	5′-GGA GCG CTA TTT CGT TCG-3′	F	878967
	5′-GTA GGC CGG GAA GCT GAT-3′	R	
*pelA*	5′-CCT TCA GCC ATC CGT TCT TCT-3′	F	878833
	5′-TCG CGT ACG AAG TCG ACC TT-3′	R	
*algA*	5′-AGA ACC AGT CGA CCT ACA TC-3′	F	879142
	5′-ACT GCA CCT CGA TGA TCT C-3′	R	

F, forward; R, reverse.
